# Cognitive Distortions, Humor Styles, and Depression

**DOI:** 10.5964/ejop.v12i3.1118

**Published:** 2016-08-19

**Authors:** Katerina Rnic, David J. A. Dozois, Rod A. Martin

**Affiliations:** a Department of Psychology, The University of Western Ontario, London, Ontario, Canada; Department of Psychology, University of Western Ontario, London, Canada

**Keywords:** cognitive distortions, humor, depression, dysphoria, negative beliefs, cognitive vulnerability

## Abstract

Cognitive distortions are negative biases in thinking that are theorized to represent vulnerability factors for depression and dysphoria. Despite the emphasis placed on cognitive distortions in the context of cognitive behavioural theory and practice, a paucity of research has examined the mechanisms through which they impact depressive symptomatology. Both adaptive and maladaptive styles of humor represent coping strategies that may mediate the relation between cognitive distortions and depressive symptoms. The current study examined the correlations between the frequency and impact of cognitive distortions across both social and achievement-related contexts and types of humor. Cognitive distortions were associated with reduced use of adaptive Affiliative and Self-Enhancing humor styles and increased use of maladaptive Aggressive and Self-Defeating humor. Reduced use of Self-Enhancing humor mediated the relationship between most types of cognitive distortions and depressed mood, indicating that distorted negative thinking may interfere with an individual’s ability to adopt a humorous and cheerful outlook on life (i.e., use Self-Enhancing humor) as a way of regulating emotions and coping with stress, thereby resulting in elevated depressive symptoms. Similarly, Self-Defeating humor mediated the association of the social impact of cognitive distortions with depression, such that this humor style may be used as a coping strategy for dealing with distorted thinking that ultimately backfires and results in increased dysphoria.

Cognitive distortions are negatively biased errors in thinking that are purported to increase vulnerability to depression (Dozois & Beck, 2008). Individuals experience automatic thoughts in response to events, which in turn lead to emotional and behavioral responses. The content of automatic thoughts is typically consistent with an individual’s core beliefs about important aspects of themselves, others, and the world. When negative core beliefs are activated and negative automatic thoughts elicited (comprised of errors in reasoning that are not evidence-based) a negative, neutral or even positive event may influence negative affect and maladaptive behaviours. Overtime, this sequence among thoughts, emotions and behaviours can cause or maintain symptoms of depression.

Cognitive distortions were first listed and described by Beck, Rush, Shaw, and Emery (1979). Burns (1980) subsequently expanded on their list and identified 10 common depressotypic thinking errors. These include mindreading (i.e., assuming that others are thinking negatively about oneself), catastrophizing (i.e., making negative predictions about the future based on little or no evidence), all-or-nothing-thinking (i.e., viewing something as either-or, without considering the full spectrum and range of possible evaluations), emotional reasoning (i.e., believing something to be true based on emotional responses rather than objective evidence), labeling (i.e., classifying oneself negatively after the occurrence of an adverse event), mental filtering (i.e., focusing on negative information and devaluing positive information), overgeneralization (i.e., assuming that the occurrence of one negative event means that additional bad things will happen), personalization (i.e., assuming that one is the cause of a negative event), should statements (i.e., thinking that things must or should be a certain way), and minimizing or disqualifying the positive (i.e., ignoring or dismissing positive things that have happened). Cognitive errors may occur with differing frequency across social and achievement domains, particularly depending on the content of an individual’s core beliefs, which typically fall into two categories: unlovability/sociotropy/dependency, or helplessness/autonomy/achievement (Beck, 1995; Beck, Epstein, Harrison, & Emery, 1983; Clark, Beck, & Alford, 1999). Given the importance of the interpersonal context for the onset and course of depression (see Evraire & Dozois, 2011; Hammen & Shih, 2014), cognitive distortions occurring in the social domain may be most relevant to depressive symptomatology.

Although cognitive distortions figure prominently in cognitive theory and therapy, a dearth of research has examined the mechanisms through which cognitive distortions impact subsequent psychological distress. Humor styles are potential mediators of the association between cognitive and interpersonal vulnerability factors and psychological dysfunction, distress, or poor interpersonal functioning (e.g., Cann, Norman, Welbourne, & Calhoun, 2008; Dozois, Martin, & Faulkner, 2013; Fitts, Sebby, & Zlokovich, 2009; Kazarian, Moghnie, & Martin, 2010; Kuiper & McHale, 2009). For example, past research has found that various humor styles mediated the relation of early maladaptive schemas (i.e., core beliefs about the self and others) and depressive symptoms (Dozois, Martin, & Bieling, 2009).

Humor styles comprise the ways in which people use humor to cope and to communicate with others. Because humor involves incongruity and can be interpreted in multiple ways, it can be used to shift perspectives regarding a stressful situation and as a way to gain a sense of mastery. Past research has indicated that some humor styles accomplish this in a way that is beneficial (Affiliative and Self-Enhancing humor), whereas other styles are maladaptive (Aggressive and Self-Defeating humor; Martin, Puhlik-Doris, Larsen, Gray, & Weir, 2003; Kuiper, Grimshaw, Leite, & Kirsh, 2004). Affiliative humor is used to facilitate relationships, amuse others, and minimize social tension through the use of spontaneous jokes, witty banter, and funny anecdotes. Self-Enhancing humor involves a humorous and cheerful outlook in life and a tendency to be amused by incongruities that facilitates emotion regulation and coping with stress and adversity. This type of humor encompasses a style of thinking, and therefore can be conceptualized as a cognitive construct. Aggressive humor is used to posture in a relationship and to demean or manipulate others through the use of sarcasm, teasing, derision, and ridicule. This style of humor involves making disparaging comments and “putting down” others in an effort to enhance one’s self, but at the expense of relationship quality. Self-Defeating humor involves excessive self-disparagement as one says or does funny things at one’s own expense in order to gain approval, amuse others, or to avoid dealing with a problem. This type of humor is ingratiating and includes allowing oneself to be the “butt” of others’ jokes. This type of humor is associated with low self-esteem and is distinct from not taking oneself overly seriously and making light of one’s faults and errors in a self-accepting manner (which would comprise Affiliative humor).

The four styles of humor, as assessed by the Humor Styles Questionnaire (Martin et al., 2003), are differentially correlated with emotional and psychosocial well-being (see Martin, 2007, for review). Self-Enhancing humor is associated with emotional well-being, including self-esteem, optimism, and positive affect, and negatively associated with depression, anxiety, rumination, perceived stress, and neuroticism. Affiliative humor, in contrast, is more closely associated with relationship variables than with emotional well-being, and is related to intimacy, relationship satisfaction, social support, interpersonal competence, secure attachment, and extraversion. Affiliative humor is inversely related to loneliness and social anxiety. Likewise, Aggressive humor is predominantly associated with relationship variables, and is negatively related to satisfaction, competence, agreeableness, and conscientiousness, and positively associated with hostility and neuroticism. Self-Defeating humor is related to anxiety, depression, anxious attachment, and neuroticism, and negatively associated with self-esteem and optimism. Whereas Self-Enhancing and low Self-Defeating humor are related to emotional well-being, Affiliative and low Aggressive humor are more closely associated with interpersonal functioning.

The aim of the current study is to investigate how both beneficial and detrimental uses of humor influence depressive symptoms. Humor styles are considered to be coping strategies, which may be used to cope with the experience of activated beliefs and distorted thinking. Because cognitive distortions involve negatively biased thinking about self and others, it was hypothesized that individuals who experience cognitive distortions frequently and whose lives are impacted by them would tend to engage in maladaptive humor styles as these would be more congruent with automatic thoughts involving themes of incompetence, worthlessness, unlovability, and assumptions that others are perceiving and thinking negatively about them. Given that these themes are relevant to low self-esteem, which Self-Defeating, not Aggressive, humor is related to, cognitive distortions may be more robustly associated with a Self-Defeating humor style. A negatively distorted type of thinking is likely not conducive to the use of Affiliative and Self-Enhancing humor (particularly in social contexts where humor is more likely to play a role), which require a sense of playfulness, the generation of positive statements, and an intention to connect with others. Furthermore, given that low Self-Enhancing and high Self-Defeating humor have been found to be most closely associated with emotional distress, these variables were hypothesized to mediate the association of cognitive distortions (both frequency and impact) with depressive symptoms. Mediating models were also tested separately for frequency and impact of cognitive distortions in interpersonal and achievement contexts in an exploratory manner.

## Method

### Participants

Participants were 208 first-year undergraduate psychology students at the University of Western Ontario, who were predominantly female (70% female, 30% male) and White (69% identified as White, 25% as Asian, 2% as Black, 1% as Hispanic, and 3% as ‘other’ or mixed race). The mean age of the sample was 18.46 (*SD* = 1.73).

### Measures

#### Cognitive Distortions Scale (CDS)

The CDS (Covin, Dozois, Ogniewicz, & Seeds, 2011) is a 20-item self-report measure that assesses the frequency of 10 types of cognitive distortions (mindreading, catastrophizing, all-or-nothing thinking, emotional reasoning, labeling, mental filtering, overgeneralization, personalization, should statements, minimizing or disqualifying the positive) across both social and achievement related (e.g., school or work) situations. Participants are presented with a definition of the distortion (referred to in the questionnaire as a ‘thinking type’ in order to reduce defensiveness) and provided with an example of that distortion in an interpersonal and achievement context. For example, for ‘should statements,’ the following definition is provided: “People sometimes think that things should or must be a certain way” and the example for interpersonal contexts is: “Anne believes that she *must* be funny and interesting when socializing.” Participants indicate the frequency with which they engage in the type of thinking on a 7-point Likert-type scale (1 = Never, 7 = All the time) in social and achievement situations. Total, social, and achievement scores are obtained by adding items. Past research has indicated that the CDS has good psychometric properties in undergraduate (Covin, Dozois, Ogniewicz, & Seeds, 2011) and clinical samples (Özdel, Taymur, Guriz, Tulaci, Kuru, & Turkcapar, 2014), including internal consistency, test-retest reliability over two weeks, and construct, discriminant, convergent, and divergent validity. In the current study, participants were also asked to rate the impact that cognitive distortions have in social and achievement contexts on a 7-point Likert-type scale (1 = Not at all, 7 = Totally). Internal consistency of each subscale was good (total frequency = .91, social frequency = .81, achievement frequency = .85, total impact = .92, social impact = .85, achievement impact = .86).

#### Humor Styles Questionnaire (HSQ)

The HSQ (Martin et al., 2003) is a 32-item self-report measure that assess adaptive (Affiliative, Self-Enhancing) and maladaptive (Aggressive, Self-Defeating) styles of humor. Sample items are: “I laugh and joke a lot with my closest friends” (Affiliative humor); “Even when I’m by myself, I’m often amused by the absurdities of life” (Self-Enhancing humor); “If someone makes a mistake, I will often tease them about it” (Aggressive humor); and “I will often get carried away in putting myself down if it makes my family or friends laugh” (Self-defeating humor). Respondents rate items by indicating the extent to which they agree with statements on a 7-point Likert-type scale (1= totally disagree; 7 = totally agree). Higher scores indicate that a particular humor style is descriptive of the participant. Past research has demonstrated good reliability and validity of subscales (Martin 2007; Martin et al., 2003), including internal consistency, test-retest reliability over one week, and construct, criterion, discriminant, and convergent validity, as well as a stable factor structure. In the current study, the internal consistency (Cronbach’s alpha) of each of the subscales was good (Affiliative = .81, Self-Enhancing = .82, Aggressive = .68, Self-Defeating = .75).

#### Beck Depression Inventory-II (BDI-II)

The BDI-II (Beck, Steer, & Brown, 1996) is a 21-item instrument that assesses the presence and severity of unipolar depressive symptoms. Individuals rate each statement on a 0 to 3 scale according to how well it describes how they have felt over the past two weeks. A sample item is “Sadness: 0 = I do not feel sad; 1 = I feel sad much of the time; 2 = I am sad all the time; 3 = I am so sad or unhappy that I can’t stand it.” Total scores are yielded by summing items, with higher scores indicating greater depressive symptoms. The BDI-II has been widely used with adult and undergraduate samples and is recognized for its strong psychometric properties, including internal consistency, test-retest reliability over several months, content, construct, criterion, convergent, and divergent validity (e.g., Dozois, Dobson, & Ahnberg, 1998; see Dozois & Covin, 2004, for a review). In the current study, internal consistency was excellent (Cronbach’s alpha = .92).

### Procedure

Participants completed measures during group testing sessions. Participants completed the CDS, HSQ, and BDI-II, as well as additional measures as part of a larger study, in randomized order. Participants were then debriefed about the nature of the study and compensated with course credit.

### Statistical Analyses

Mediation analyses were conducted to test the hypothesis that humor styles mediate the relationship between cognitive distortions and depressive symptoms. Simple correlations between the predictor variables (cognitive distortions), mediator variables (humor styles) and the criterion variable (depressive symptoms) were first examined (see Table 1). A prerequisite for mediation is that all correlations between a predictor and mediator, mediator and criterion, and predictor and criterion for a given analysis be significant (Baron & Kenny, 1986). Mediation analyses were conducted only for the cognitive distortions and corresponding mediators that met this requirement. To test for the potential mediating effects of humor styles, the bootstrap sampling procedure developed by Preacher and Hayes (2008) was used. This procedure examines and tests the direct effect of the predictor variable on the criterion variable and the indirect (i.e., mediating) effect through the pathway of the mediator variable. The bootstrap procedure uses sampling with replacement to draw a large number of samples (1,000 in the present study) from the data set, and path coefficients are calculated for each sample. Using estimates based on the 1,000 samples, the mean direct and indirect effects and their confidence intervals (CIs) are computed. These CIs are used to determine whether or not an effect is statistically significant. For each effect, the corresponding Bias Corrected 95% or 99% CI was examined; if the range did not cross zero, the effect was considered significant at the .05 or .01 level, respectively. An advantage of the bootstrap-driven approach is that it does not assume a normal distribution of variables, unlike product-of-coefficient approaches such as the Sobel test.

All mediation analyses were conducted using the macro provided by Preacher and Hayes (2008) for conducting the bootstrap procedure. Note that in the figures and tables presented, path coefficients and corresponding *p*-values are based on mediation analyses without bootstrapping, since the bootstrapping procedure only provides Bias Corrected CIs in the output. Because the bootstrapping procedure provides a more robust analysis, the evaluations of significance in the analyses below are based on bootstrapping. All variables in the analysis were standardized (*M* = 0, *SD* = 1.0), to allow for a comparison of results across analyses. Path coefficients can therefore be interpreted in a manner similar to correlation coefficients.

## Results

The means and standard deviations for the six CDS subscales, the four HSQ subscales, and the BDI-II are presented in Table 2 for descriptive purposes. Pearson correlations between the CDS scales, HSQ scales, and BDI-II are presented in Table 1. Cognitive Distortion Frequency was significantly negatively correlated with Affiliative and Self-Enhancing humor. This variable was also positively associated with Aggressive and Self-Defeating humor. The same pattern of correlations was found for Cognitive Distortion Social Frequency. Cognitive distortion Achievement Frequency was significantly and negatively related to Self-Enhancing humor, and positively correlated with Self-Defeating humor. Cognitive Distortion Impact was negatively correlated with Affiliative and Self-Enhancing humor, and positively correlated with Self-Defeating humor. The same pattern of correlations was found for Cognitive Distortion Social and Achievement Impact.

**Table 1:** Pearson Correlation Coefficients Between Depressive Symptoms, Cognitive Distortions, and Humor Styles

Scale	BDI-II	HSQ-Affiliative Humor	HSQ-Self-Enhancing Humor	HSQ-Aggressive Humor	HSQ-Self-Defeating Humor
CDS-Frequency	.35***	-.16*	-.16*	.17*	.31***
CDS-Social Frequency	.33***	-.17*	-.20*	.17*	.39***
CDS-Achievement Frequency	.31***	-.13	-.10*	.14	.29***
CDS-Impact	.31***	-.20*	-.22**	.03	.23**
CDS-Social Impact	.31***	-.19*	-.25**	.04	.21**
CDS-Achievement Impact	.27**	-.18*	-.16*	.03	.22**
HSQ-Affiliative Humor	-.11				
HSQ-Self-Enhancing Humor	-.23**				
HSQ-Aggressive Humor	.18**				
HSQ-Self-Defeating Humor	.28***				

*Note.* BDI-II = Beck Depression Inventory-II; CDS = Cognitive Distortions Scale; HSQ = Humor Styles Questionnaire.

*p < .05. **p < .01. ***p < .001.

**Table 2:** Descriptive Statistics for Cognitive Distortions, Humor Styles, and Depressive Symptoms

Variable	*M*	*SD*
CDS-Frequency	81.00	18.16
CDS-Social Frequency	41.60	9.27
CDS-Achievement Frequency	39.41	10.21
CDS-Impact	80.96	19.15
CDS-Social Impact	41.82	9.97
CDS-Achievement Impact	39.14	10.60
HSQ-Affiliative Humor	47.63	6.39
HSQ-Self-Enhancing Humor	36.28	8.48
HSQ-Aggressive Humor	29.61	7.31
HSQ-Self-Defeating Humor	26.76	8.08
BDI-II	9.79	6.53

*Note.* BDI-II = Beck Depression Inventory-II; CDS = Cognitive Distortions Scale; HSQ = Humor Styles Questionnaire.

All scales of the CDS were significantly positively correlated with the BDI-II, consistent with the idea that cognitive distortions are vulnerability factors for dysphoria and depression. Furthermore, consistent with past research, the BDI-II was negatively correlated with Self-Enhancing humor, and positively associated and Self-Defeating humor. It was also positively related to Aggressive humor.

**Figure 1:**
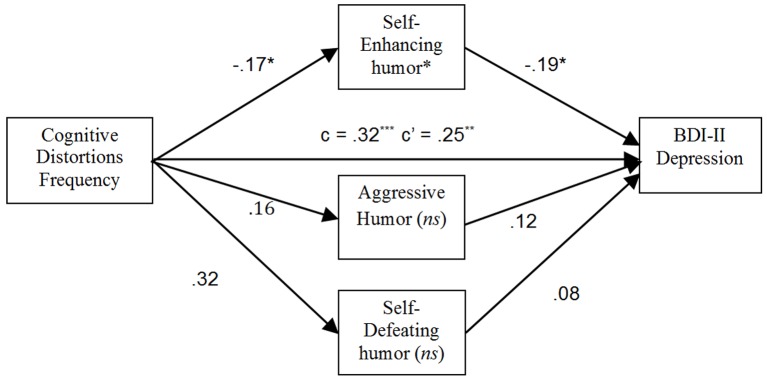
Mediating effect of Self-Enhancing humor on the relation between frequency of cognitive distortions and depressive symptoms. *p < .05. **p < .01. ***p < .001. *ns* = Not statistically significant.

Multiple mediation analyses were conducted using the procedure described earlier to examine potential mediating effects of the humor styles on the relationships between each of the CDS subscales and the BDI-II. In the analysis using CDS Frequency as the predictor variable, HSQ Self-Enhancing, Aggressive, and Self-Defeating humor styles were included as potential mediators, as these were the only humor styles correlated with both CDS Frequency and BDI-II scores. Results of this analysis are presented in Figure 1. A significant mediating effect was found for Self-Enhancing humor (*p* < .05), but the mediating effects for Aggressive and Self-Defeating humor were not significant. Therefore, higher scores on CDS Frequency were associated with lower Self-Enhancing humor, which in turn predicted higher BDI-II scores. In addition to the indirect effect of CDS Frequency on dysphoria through Self-Enhancing humor, a direct effect was also found (*p* < .01), indicating that Self-Enhancing humor only partially mediated this relationship.

**Figure 2:**
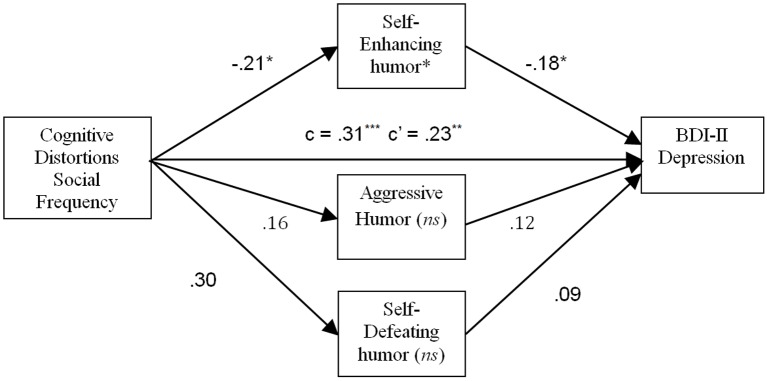
Mediating effect of Self-Enhancing humor on the relation between frequency of cognitive distortions in social contexts and depressive symptoms. *p < .05. **p < .01. ***p < .001. *ns* = Not statistically significant.

In the mediation analysis for CDS Social Frequency, HSQ Self-Enhancing, Aggressive, and Self-Defeating humor styles were again included as potential mediators as determined by the pattern of correlations found earlier. The results are shown in Figure 2. In this analysis, and similar to the analysis for CDS Frequency, a significant mediating effect was found for Self-Enhancing humor (*p* < .05), but the mediating effects for Aggressive and Self-Defeating humor were not significant. Therefore, higher scores on CDS Social Frequency were associated with lower Self-Enhancing humor, which in turn predicted higher BDI-II scores. Furthermore, in addition to the indirect effect of CDS Frequency on dysphoria through Self-Enhancing humor, a direct effect was also found (*p* < .01), indicating that Self-Enhancing humor only partially mediated this relationship.

**Figure 3:**
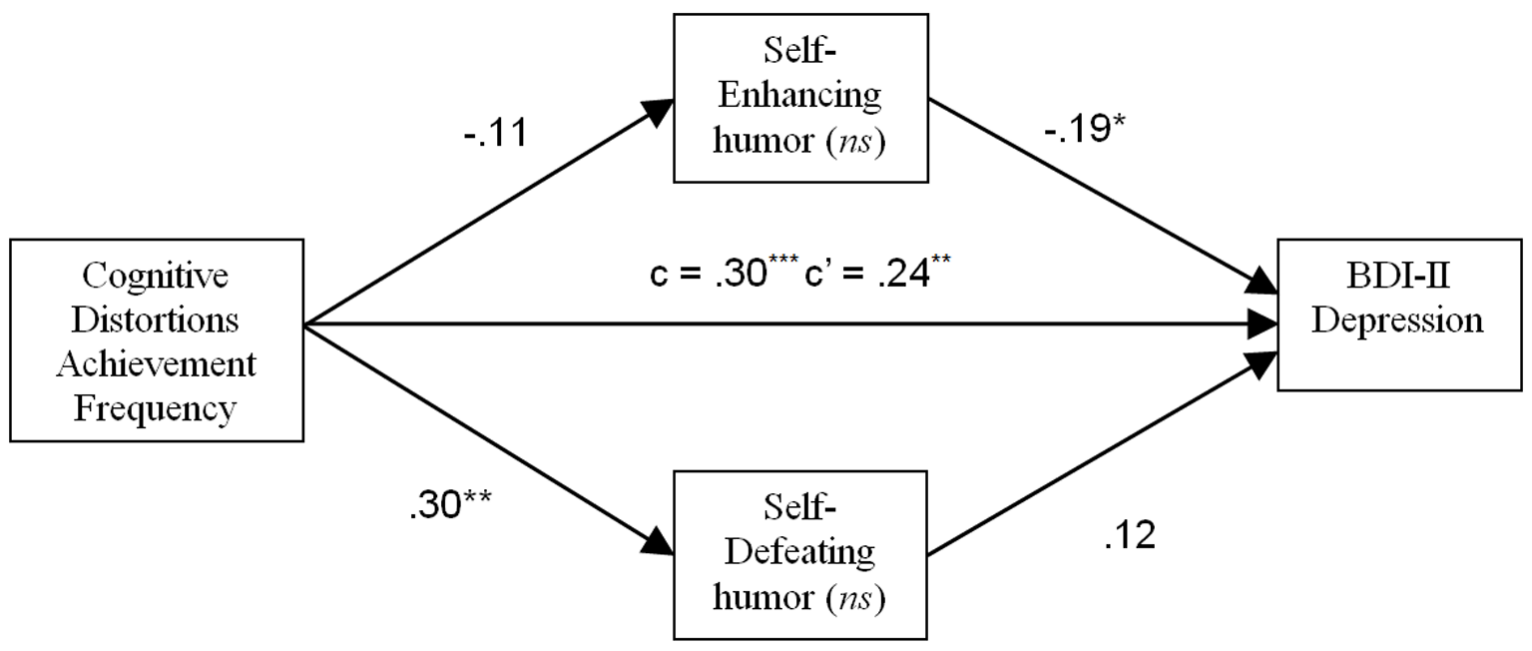
Nonsignificant mediating effect of humor styles on the relation between frequency of cognitive distortions in achievement contexts and depressive symptoms. *p < .05. **p < .01. ***p < .001. *ns* = Not statistically significant.

In the analysis using CDS Achievement Frequency as the predictor variable, only HSQ Self-Enhancing and Self-Defeating humor styles were included as potential mediators based on the obtained pattern of correlations. The results are presented in Figure 3. No significant mediating effects were found, however there was a direct effect of CDS Achievement Frequency on dysphoria (*p* < .01).

**Figure 4:**
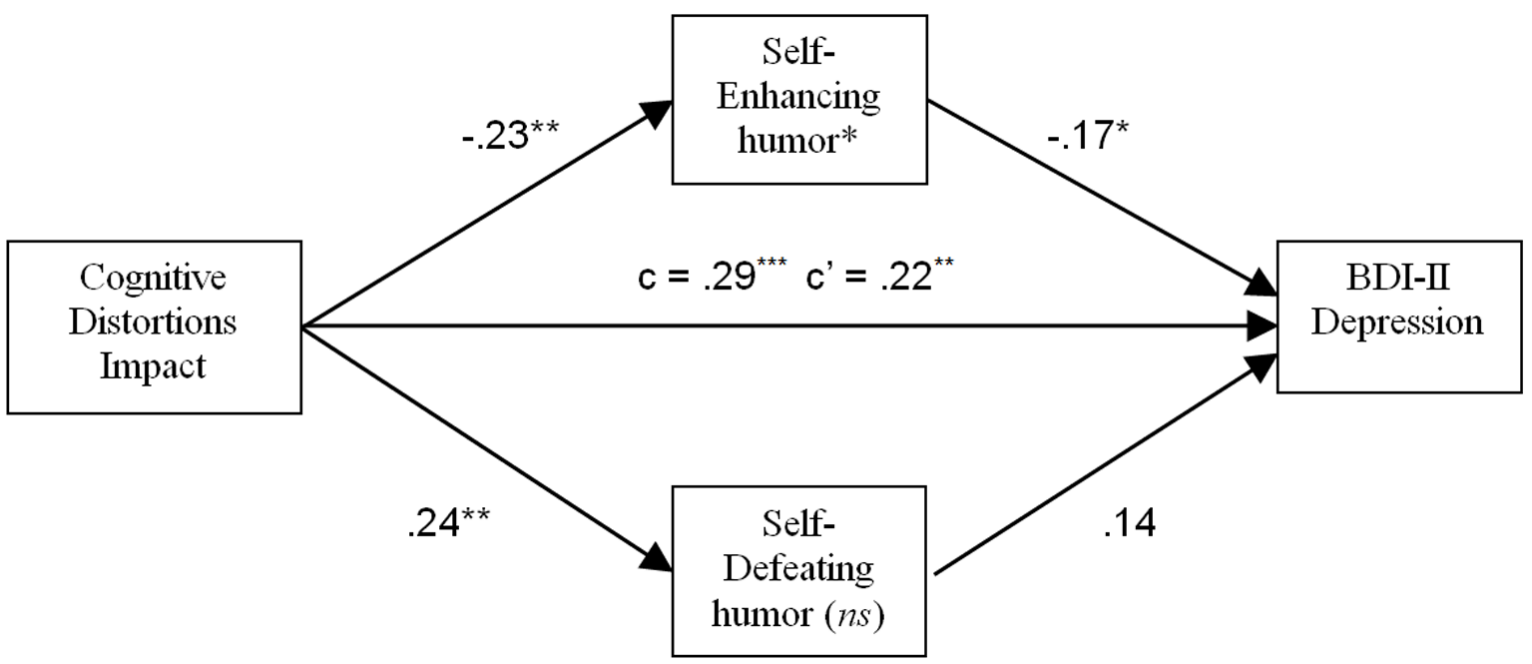
Mediating effect of Self-Enhancing humor on the relation between impact of cognitive distortions and depressive symptoms. *p < .05. **p < .01. ***p < .001. *ns* = Not statistically significant.

In the analysis using CDS Impact as the predictor, HSQ Self-Enhancing and Self-Defeating humor styles were included as potential mediators, see Figure 4. A significant mediating effect was found for Self-Enhancing humor (*p* < .05), and the mediating effect for Self-Defeating humor was nonsignificant. Higher scores on CDS Impact predicted lower Self-Enhancing humor, which in turn predicted higher BDI-II scores. The direct effect of CDS Impact on dysphoria was also significant, (*p* < .01), indicating a partial mediating effect of Self-Enhancing humor.

**Figure 5:**
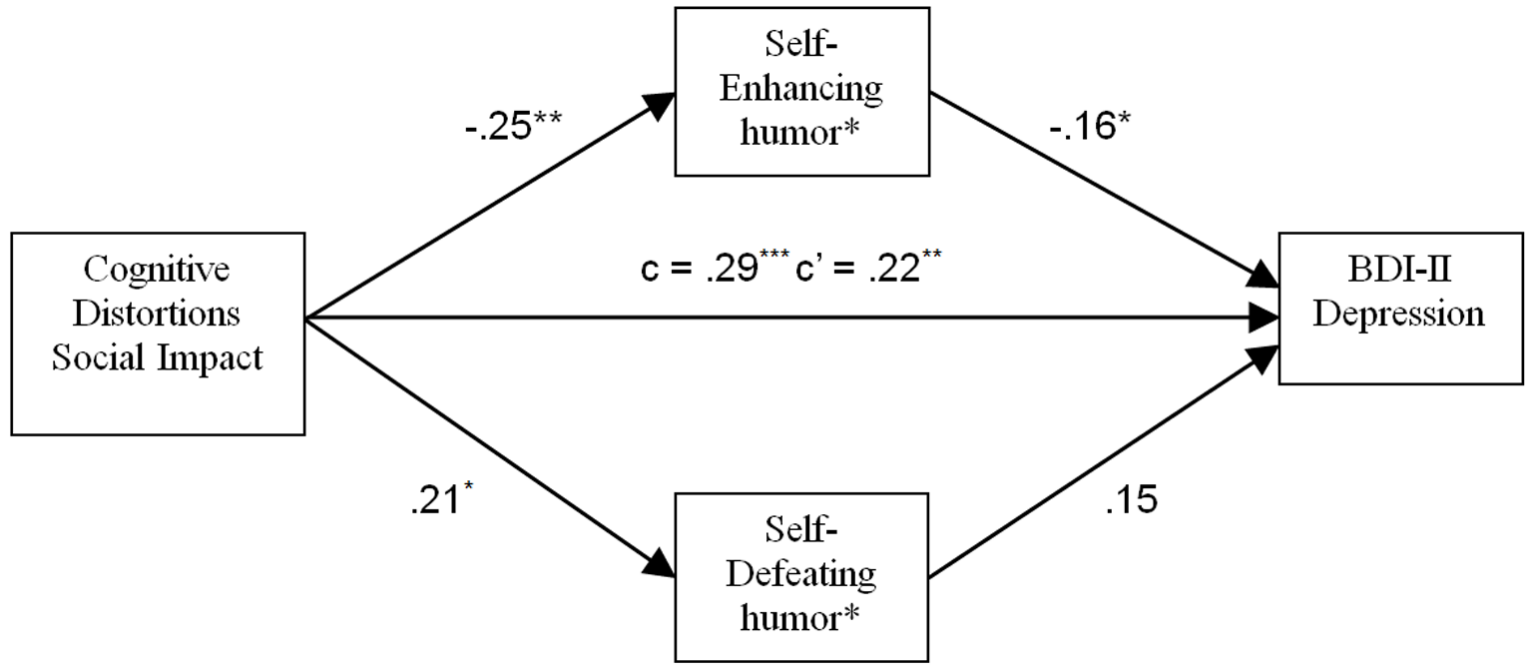
Mediating effect of Self-Enhancing and Self-Defeating humor on the relation between impact of cognitive distortions in social contexts and depressive symptoms. *p < .05. **p < .01. ***p < .001.

Figure 5 shows the results of the analysis for CDS Social Impact, in which we entered Self-Enhancing and Self-Defeating humor as potential mediators as determined by the pattern of simple correlations. In this analysis, significant mediating effects were found for both Self-Enhancing (*p* < .05) and Self-Defeating humor (*p* < .05). Higher scores on CDS Social impact predicted lower scores on Self-Enhancing and higher scores on Self-Defeating humor, which in turn predicted higher BDI-II scores. The direct effect of CDS Social Impact on dysphoria was significant (*p* < .01), indicating that the humor styles were only partial mediators.

**Figure 6:**
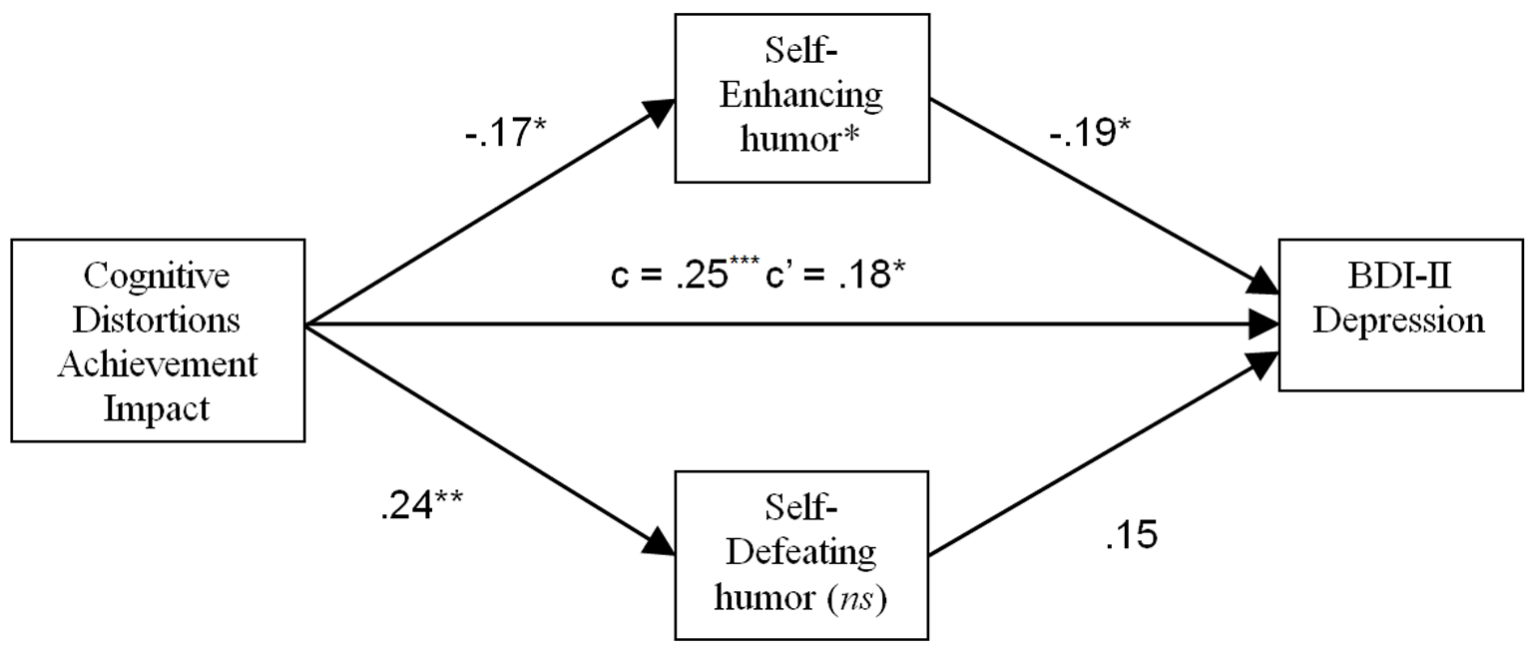
Mediating effect of Self-Enhancing humor on the relation between impact of cognitive distortions in achievement contexts and depressive symptoms. *p < .05. **p < .01. ***p < .001. *ns* = Not statistically significant.

Finally, in the analysis for CDS Achievement Impact, Self-Enhancing and Self-Defeating humor were entered as potential mediators. Only Self-Enhancing humor mediated the relation of CDS Achievement Impact with depressive symptoms (*p* < .05). No mediating effect was found for Self-Defeating humor, see Figure 6. Therefore, higher scores on CDS Achievement Impact predicted lower Self-Enhancing humor, which in turn predicted higher BDI-II scores. The direct effect of CDS Achievement Impact on dysphoria was also significant, (*p* < .05), indicating that Self-Enhancing humor was only a partial mediator.

## Discussion

The purpose of the current study was to examine the relationships among cognitive distortions, adaptive and maladaptive humor styles, and depressive symptoms. Another objective was to evaluate whether humor styles mediate the relationship between cognitive distortions and dysphoria. As predicted, the frequency and impact of cognitive distortions, as well as frequency and impact ratings of cognitive distortions in both interpersonal and achievement-related contexts, were positively and significantly associated with depressive symptomatology. This finding is consistent with cognitive theory, which posits that cognitive distortions are a form of automatic thoughts related to negative affect and depressed mood. This finding is also supportive of the practice of assessing and altering cognitive distortions as a treatment target in cognitive behavioral therapy for depressed individuals (see Clark, 2014).

Consistent with past research, self-reported depressive symptoms were negatively related to use of Self-Enhancing humor and positively associated with Self-Defeating humor. However, inconsistent with past research, dysphoria was not related to Affiliative humor in the present study, although pearson’s *r* was in in the expected direction (i.e., negative). Past research has found modest negative correlations of Affiliative humor with dysphoria (Dozois et al., 2009; Frewen, Brinker, Martin, & Dozois, 2008) using an undergraduate sample that was similar in terms of age and gender distribution. However, the current sample exhibited a smaller mean and standard deviation in BDI-II scores. It is possible that the current sample lacked sufficient severity and variance in self-reported depressive symptoms to detect a true correlation between dysphoria and Affiliative humor. Moreover, Affiliative humor is both theoretically and empirically more pertinent to relational functioning than to emotional well-being (Martin, 2007), so it is not entirely surprising that a significant relationship was not found in the current study. Additionally, and also in contrast with past research, the current study found that dysphoria was associated with an Aggressive humor style (e.g., Dozois et al., 2009; Frewen et al., 2008; see Martin, 2007 for review). This is consistent with the finding that depression is predictive of greater generation of interpersonal conflict (i.e., interpersonal stress generation; Hammen, 1991), such that some of this conflict may arise from the depressed individual’s use of Aggressive humor.

Consistent with hypotheses, cognitive distortions were inversely associated with beneficial styles of humor, and positively correlated with detrimental humor styles. Affiliative humor was significantly and negatively associated with all forms of cognitive distortions, with the exception of frequency of cognitive distortions in achievement situations, which was not associated with Affiliative humor. This is likely because achievement contexts are less relevant for efforts to affiliate with others. Affiliative humor is used to facilitate relationships and reduce tension, and involves a sense of playfulness and spontaneity. Negatively distorted thinking, in contrast, involves a rigid pattern of thinking that lacks the openness, flexibility, and creativity required to generate funny comments and anecdotes. Moreover, when individuals are thinking negatively, they are less able to access and retrieve information and memories that are incongruent with their current negative state (Bower, 1981; Ingram, Steidtmann, & Bistricky, 2008). Moreover, depending on the content of negative distortions, individuals may be assuming that others do not think well of them or will not respond favorably to their efforts to be humorous. If the content of their thinking is self-focused, they may lack the self-efficacy and confidence to make a joke in an effort to bond with others or ‘lighten the mood.’ Similarly, all types of cognitive distortions were associated with reduced Self-Enhancing humor. This finding is likely due to similar reasons as above. Furthermore, it is likely difficult to adopt a humorous, cheerful perspective and to feel amusement when the valence and content of one’s thoughts are in direct conflict. As a predominantly cognitive construct, Self-Enhancing humor is unlikely to coincide with negative cognitive distortions. Aggressive humor was only associated with overall frequency and social frequency of cognitive distortions, such that greater frequency of cognitive distortions was related to increased use of Aggressive humor. Cognitive distortions may involve thoughts that others are hostile or have bad intentions, leading the individual to engage in Aggressive humor out of defensiveness or to retaliate for perceived hostility. The finding that Aggressive humor was not associated with other facets of cognitive distortions is consistent with past research that Aggressive humor is not related to self-esteem (Martin et al., 2003), a construct that represents negatively biased views of the self, and therefore has overlap with cognitive distortions. It is possible that use of Aggressive humor is driven more by other variables, such as hostility. Finally, Self-Defeating humor was positively associated with all forms of cognitive distortions. Individuals who engage in biased negative thinking about themselves may attempt to use humor to gain the approval of others to feel better but, because the content of their thoughts is negative, are more likely to retrieve negative self-relevant information and beliefs, and therefore generate humor that is consistent and is therefore self-disparaging.

An additional goal of this study was to examine whether humor styles mediate the relation of cognitive distortions with dysphoria. Humor styles can be conceptualized as emotion regulation, coping, and communication strategies that are likely influenced by the frequency and impact of cognitive distortions and that may in turn influence severity of depression. Multiple mediation analyses indicated that humor styles partially mediated all types of cognitive distortions, with the exception of the frequency of cognitive distortions in achievement-relevant situations. It is possible that other, less socially relevant behaviours play a role in influencing dysphoria in these contexts, such as procrastination, avoidant coping, and rumination. All other cognitive distortions (overall frequency, frequency in social contexts, overall impact, and impact in social and achievement contexts) were mediated by Self-Enhancing humor, such that cognitive distortions predicted reduced use of Self-Enhancing humor, which in turn predicted greater depressive symptoms. This finding suggests that the experience of negative thinking may interfere with an individual’s ability to adopt a humorous and cheerful outlook on life (i.e., use Self-Enhancing humor) as a way of regulating emotions and coping with stress, thereby resulting in elevated depressive symptoms. In addition, use of Self-Defeating humor (along with decreased Self-Enhancing humor), partially mediated the relation of the impact of cognitive distortions in social situations with dysphoria. Therefore, individuals who experience cognitive distortions in social situations may respond to these thoughts by attempting to connect with others by making jokes at their own expense. This strategy backfires, however, and results in increased dysphoria. The use of Self-Defeating humor may reinforce the individual’s negative self-concept (thereby increasing negative affect), especially when others appear to agree with the individual’s humorous actions or statements, or to react to their use of humor in a rejecting manner. Altogether, these findings are consistent with past research, which found that Self-Enhancing and Self-Defeating humor styles are more predictive of emotional well-being and distress, including depression, than are Affiliative and Aggressive humor, variables for which mediation was not found. Among the positive humor styles, Self-Enhancing humor is more relevant to cognition, whereas Affiliative humor is more relevant to relationships, which could explain why Self-Enhancing humor plays a role in the relationship of cognitive distortions and depression. Among the negative humor styles, Self-Defeating humor is related to self-esteem, a variable associated with both cognitive distortions and depression, whereas Aggressive humor is not, which similarly could explain why Self-Defeating humor was the only maladaptive humor style to demonstrate mediating effects.

A limitation of this study was its cross-sectional design. We cannot rule out, based on the current data, whether humor styles actually precede the tendency to engage in distorted thinking, that prior depression predicts cognitive distortions and use of humor styles, or that an unmeasured third variable accounts for both cognitive distortions and humor style. Moreover, current depressive symptomatology may have influenced the use or reporting of humor styles. Although temporal precedence cannot be determined based on the current study, results are consistent with the hypothesis that the association of cognitive distortions and dysphoria is at least partially mediated by reduced use of adaptive humor and, in one case, increased use of maladaptive humor, to cope with stress. In addition, the generalizability of the study is limited by the nature of the sample, which was predominantly comprised of young adult Caucasian females. Whether the same results would be obtained in a clinical sample (e.g., individuals with major depression) with greater endorsement of cognitive distortions and depressive symptoms, or in a sample with a greater proportion of males, is an empirical question for future research. Longitudinal research is needed to examine changes in the relationships between cognitive distortions, humor styles, and depression across the lifespan. Moreover, future research should examine the relation of cognitive distortions with humor styles and depressive symptoms using behavioural and process measures. An examination of whether information processing influences the ability to use various styles of humor when an individual has recently engaged in distorted thinking is another question worthy of further study.

This study demonstrates that cognitive distortions, which represent a cognitive vulnerability to depression, are mediated by low use of an adaptive humor style, Self-Enhancing humor (with the exception of frequency of cognitive distortions in achievement-related contexts) and, in one case (social impact of cognitive distortions), use of a maladaptive humor style (i.e., Self-Defeating humor). Mediating effects were not found for Affiliative and Aggressive humor. Furthermore, these findings add to the already extensive literature that supports the discriminant and construct validity of the four scales of the HSQ (Martin, 2015; see Heintz & Ruch, 2015). From a treatment perspective, increasing use of adaptive coping strategies for managing stressful situations (in particular use of Self-Enhancing humor) and decreasing use of maladaptive strategies may be useful for individuals experiencing depressive symptoms. Targeting cognitive distortions themselves in order to shift an individual toward more evidence-based thinking may also be useful in altering use of humor and decreasing depressive symptomatology. However, future research is needed to examine these questions and to determine the specific mechanisms through which cognitive distortions and humor styles confer risk for depression.
